# Does exogenous hormonal therapy affect the risk of glioma among females: A systematic review and meta-analysis

**DOI:** 10.1093/noajnl/vdad167

**Published:** 2023-12-23

**Authors:** Ghady Alfuridy, Rana Alghamdi, Abdulaziz Alkhoshi, Ahood Mahjari, Abdullah Alhussein, Ebtihaj Alshehri, Ahmed Lary, Abdulrahman Sabbagh, Soha Alomar

**Affiliations:** College of Medicine, Faculty of Medicine, King Abdulaziz University, Jeddah, Saudi Arabia; College of Medicine, Faculty of Medicine, King Abdulaziz University, Jeddah, Saudi Arabia; College of Medicine, Faculty of Medicine, King Abdulaziz University, Jeddah, Saudi Arabia; College of Medicine, Faculty of Medicine, Najran University, Najran, Saudi Arabia; College of Medicine, Faculty of Medicine, King Abdulaziz University, Rabigh, Saudi Arabia; College of Medicine, Faculty of Medicine, King Khalid University, Abha, Saudi Arabia; Division of Neurosurgery, Department of Surgery, Faculty of Medicine, King Abdulaziz Medical City, Jeddah, Saudi Arabia; Division of Neurosurgery, Department of Surgery, Faculty of Medicine, King Abdulaziz University, Jeddah, Saudi Arabia; Division of Neurosurgery, Department of Surgery, Faculty of Medicine, King Abdulaziz University, Jeddah, Saudi Arabia

**Keywords:** brain tumors, glioma, hormonal replacement therapy, hormonal therapy, oral contraceptive pills

## Abstract

**Background:**

The effect of exogenous hormone replacement therapy (HRT) and oral contraceptive pills (OCPs) on glioma risk in females is unclear despite numerous studies; hence, we conducted a meta-analysis to evaluate this relationship.

**Methods:**

Studies investigating the impact of exogenous female hormones on glioma risk were retrieved by searching 4 databases from inception until September 2022. Articles of any design, such as case–control and cohort studies, proving the relative risk (RR), odds ratio (OR), or hazard ratio were included. Summary OR values were calculated using a random effects model.

**Results:**

Both HRT and OCP use of any duration decreased the risk of developing glioma [HRT OR = 0.78, 95% CI 0.66–0.91, *P* = .00; OCP: OR = 0.80, 95% CI 0.67–0.96, *P* = .02]. When stratified by duration of use, HRT use >1 year significantly reduced glioma risk (<1 year: OR = 0.82, 95% CI 0.63–1.07, *P* = 0.15; 1–5 years: OR = 0.79, 95% CI 0.67–0.92, *P* = .00; 5–10 years: OR = 0.80, 95% CI 0.66–0.97, *P* = .02; >10 years: OR = 0.69, 95% CI 0.54–0.88, *P* = .00). In contrast, only OCP use for >10 years significantly reduced glioma risk (<1 year: OR = 0.72, 95% CI 0.49–1.05, *P* = .09; 1–5 years: OR = 0.88, 95% CI 0.72–1.02, *P* = .09; 5–10 years: OR = 0.85, 95% CI 0.65–1.1, *P* = 0.21; >10 years: OR = 0.58, 95% CI 0.45–0.74, *P* = .00).

**Conclusions:**

Our pooled results strongly suggest that sustained HRT and OCP use is associated with reduced risk of glioma development.

**Key Points**

- Hormone replacement therapy (HRT) for 1 year or more is associated with reduced glioma risk.
- Oral contraceptive use for 10 years or more is associated with reduced glioma risk.

**Importance of the Study**

This updated meta-analysis and systematic review reveals a significant association between hormonal therapy and reduced glioma risk among adult females. These findings may warrant further evaluation of the role of female hormones as preventative therapies for glioma.

Gliomas are among the most common primary tumors in the CNS. These tumors are usually classified according to histological features as diffuse glioma, circumscribed astrocytic glioma, glioneuronal and neuronal tumor, ependymomas, and a handful of rare cases of no clear histological class.^1^ The global incidence of brain tumors is estimated to be 10.82 per 100 000 person-years, and these neoplasms account for 2.71% of all cancer-related deaths.^2^ The only 2 confirmed risk factors for glioma are ionizing radiation and hereditary syndromes such as neurofibromatosis 1 and 2, tuberous sclerosis, Lynch syndrome, and von Hippel–Lindau syndrome.^2^ The incidence of glioma is also higher among males, suggesting that development may be influenced by hormones.^3^ Consistent with this notion, glioma cells express steroid hormone receptors^4^ and factors such as duration of exogenous hormone use, age at first childbirth, number of births, age at menarche, age at menopause, and type of menopause (natural or medically induced), and duration of hormone alter glioma incidence.^5^ There are many important indications for hormone replacement therapy (HRT), including treatment of menopause symptoms and prevention of cardiovascular disease or osteoporosis.^6^ Hot flashes and urogenital atrophy are common examples of postmenopausal symptoms that are frequently managed by HRT.^7^ It was reported that 44% of postmenopausal females have used HRT at least once, most often in pill form (40%).^8^

While numerous studies have addressed the effects of HRT and oral contraceptive pills (OCPs) on glioma risk, many of the results are contradictory. For instance, Benson et al. reported an increased risk of developing glioma and meningioma,^9^ while Yang et al. found that risks of glioma and meningioma were dependent on the duration of OCP use.^10^ Others have found that factors such as old age at menarche increase the risk of developing glioma.^11,12^ Conversely, Lan et al. reported that HRT reduced the risk of developing glioma, although they did not stratify by duration of use.^13^ In this meta-analysis, we examined the relationship between glioma risk and the use of HRT or OCP with duration of use stratification.

## Methods

### Search Strategy

This study was conducted according to Preferred Reporting Items for Systematic Reviews and Meta-Analysis (PRISMA) guidelines.^14^ Studies on the effects of HRT on glioma risk in females were retrieved by searching Medline, Cochrane, Embase, and CENTRAL as well as the references lists of included papers and previous meta-analyses. Searches were conducted in September 2022 and were restricted to English language literature. The search string used for all databases was as follows: [(Brain Glioma OR high-grade tumor OR glial cell neoplasm OR glioblastoma multiforme OR GBM OR diffuse glioma OR glial tumors OR anaplastic glioma) AND (hormone replacement therapy OR contraceptives OR exogenous hormones OR exogenous estrogen OR estrogen OR HRT OR OCP) AND (risk OR health risk assessment OR risk factor)].

### Study Selection

Inclusion criteria were: (i) studies describing the relationship between glioma incidence and current or past use of female exogenous hormones using a case–control or cohort study design, and (ii) providing the relative risk (RR), odds ratio (OR), or hazard ratio. No randomized controlled trials were identified through our search. Retrieved studies conducted in animal models, presented as conference abstracts, that did not classify CNS tumor subtypes or did not include glioma as the outcome of interest were excluded. In addition, reviews and previous meta-analyses were excluded. Two groups of authors independently performed the primary survey according to our preset inclusion criteria, and conflicts were resolved by senior authors through discussion and consensus.

### Data Extraction and Quality Assessment

The following parameters were extracted from each study and entered into an Excel sheet: first author, year of publication, country where the study was conducted, mean or median age, sample size, study design, follow-up duration, exposure (HRT, OCPs, or both), risk estimate, duration of use, and Newcastle—Ottawa Scale (NOS). The data were then reviewed by a third author.

Study quality was assessed using the NOS, a well-validated metric for evaluating observational and nonrandomized studies according to participant selection criteria, comparability, and exposure or outcome. Comparability points were given whenever the age at glioma diagnosis and duration of hormone use were available. Additionally, the adequacy of the follow-up duration was determined by the senior authors. The NOS score ranges from 0 to 9 stars, and studies with≥6 stars are considered to be of relatively higher quality.^15^ We searched for the source of funding and reported it as yes (provided), no (not provided), or not mentioned (Table 1).

**Table 1.** Summary of Included Studies

First Author	Publication Year	Country	Age (Mean or Median in y)	*N*	Study Design (CC, C)	Follow-Up Duration (Mean or Median)	Exposure	Duration of Use	Source of Funding	NOS
Krishnamachari^5^	2014	United States	Mean: 51.4	968 cases 1322 controls	CC	N/A	OCP, HRT	Divided into: under 1 y, 1–5 y, 6–10 y, >10 y	Yes	7/9
Andersen^16^	2015	Denmark	Median 32	317 cases 2126 controls	CC	N/A	Hormonal contraceptive	Divided into: <1 y, 1 to less than 5 y, 5 y or more	Yes	5/9
Andersen^17^	2013	Denmark	Median 69.5	658 cases 4350 controls	CC	N/A	HRT	Divided into: <1, 1 to <5, 5 to <10, >10 y	Yes	5/9
Hatch^18^	2005	United States	Mean 51.8	212 cases 436 controls	CC	N/A	OCP and HRT	Divided into: never, <1, 1–4, 5–9, >10 y	Not mentioned	7/9
Huang^19^	2004	United States	Mean 52	341 cases 527 controls	CC	N/A	OCP and HRT	OCP and HRT: ≤5, >5	Yes	6/9
Anic^20^	2014	United States	Median 54	507 case 695 controls	CC	N/A	OCP and HRT	<1 y, 1–9 y, <9 y	Yes	8/9
Wigertz^21^	2006	Sweden	Median 44.5	115 cases 323 controls	CC	N/A	OCP, HRT, and other hormonal contraceptive	OCP & HRT: <1, 1–4, 5–9, ≥10 y	Yes	7/9
Kabat^22^	2011	United States	Median 60.6	225 355 cases	C	7.5 y	OCP and HRT	Divided into: OCP: ever, HRT: never, <5, 5–9, >10	Not mentioned	8/9
Michaud^23^	2010	Europe	Mean 54.1	276 212 cases	C	8.4 y	OCP, HRT	Divided into: OCP: never, <1,1–5, 5–10, 10–15, >15 HRT: never and <1, 1–3, 3–5, 5–10, >10	Yes	8/9
Silvera^24^	2006	Canada	Mean 60	89830 cases	C	16.4 y	OCP, HRT	OCP: 1–11 months, 12–35 months, 36–71 months, 72 months HRT: 1–35 months, 36 months	Yes	8/9
Benson^25^	2010	United Kingdom	Mean 56.6	1 147 894 cases	C	5.3 y	HRT	past use, <5 y, 5 y or more	Yes	8/9
Felini^26^	2008	United States	Mean 56.3	619 cases 650 controls	CC	N/A	OCP, HRT	<1 y, 1–4 y, 5–9 y, 10 y	Yes	7/9
Wang^27^	2011	United States	Not mentioned	357 cases 822 controls	CC	N/A	OCP, HRT	Ever use	Yes	6/9
Benson^28^	2008	United Kingdom	Mean 55.9	1 249 670 cases	C	6,2	OCP	Never, <5 y, 5+	Yes	
Hochberg^29^	1990	United States	Not mentioned	160 cases 128 controls	CC	N/A	OCP	Ever used	Yes	6/9
Benson^9^	2014	United Kingdom	50–79	689 cases 2756 controls	CC	Observation mean 8.5 y	HRT	<5 y, >5y	Yes	6/9
Schlehofer^30^	1999	United Kingdom, Australia, France, Sweden, Canada, German	Mean 42.4	1178 cases 2493 controls	CC	N/A	Steroid hormones	Not mentioned	Yes	8/9

CC = case–control, C = cohort, NOS = New castle–Ottawa scale.

### Analysis

Descriptive statistics, including mean and frequency, were calculated using IBM SPSS version 2, while the meta-analysis was conducted using Comprehensive Meta-Analysis software version 3. Summary ORs) and RR with 95% CI of developing glioma were calculated separately. Due to the rarity of glioma, ORs were considered equivalent to RRs. For simplicity, therefore, pooled results are expressed as ORs. The influences of oral contraceptives and HRT on glioma risk were also examined separately. Additional subgroup analyses were performed on treatment groups stratified by duration of use (when available) as follows: <1 year, 1–5 years, 5–10 years, and >10 years. A study design influences the risk of bias, this assessment was conducted separately for case–control and cohort studies. The possibility of heterogeneity was evaluated using the *I*-squared statistic, with <25% considered low, 25–50% moderate, and 50–75% as high heterogeneity. Due to the heterogeneity among studies, a random effects model was for pooled analysis. Sensitivity analysis was performed by omitting 1 study at a time and assessing the stability of the result and by omitting studies with NOS scores less than 6. Publication bias was assessed using Begg’s funnel plot and Egger’s test.

## Results

### Search Results and Study Characteristics

**Figure 1.**
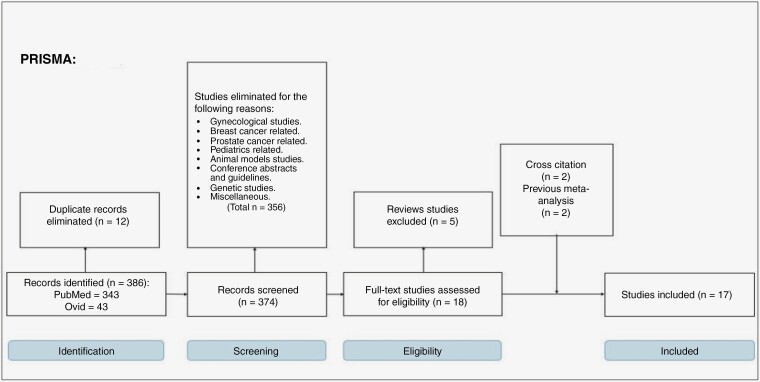
PRISMA flow chart for the search strategy.

A total of 386 studies were retrieved from Midline, Cochrane, Embase, or CENTRAL using the indicated search string. Among these, 12 were excluded as duplicates and 356 due to irrelevance after reviewing the title and abstract. The full texts of the remaining 18 studies were carefully examined, and 5 was excluded as reviews. However, 4 studies found by searching the reference lists of included studies (*n* = 2) and previous meta-analysis (*n* = 2) were included. Finally, 17 valid observational studies were enrolled, 12 population-based case–control studies^5,9,16–21,26,27,29,30^ and 5 cohort studies^22–25,28^ (Figure 1). The basic features of the enrolled studies are summarized in Table 1. Among the 17 observational studies included, 4 examined the effect of OCPs on glioma risk, 3 examined the effect of HRT, and 10 examined the effects of both HRT and OCPs.

### Descriptive Statistics and Participant Demographics

The secondary aim of this study was to provide updated descriptive statistics on glioma and associations with OCP and HRT use. The 17 studies included in this meta-analysis were conducted in 5 different countries, of which the United States of America was the site of the greatest number. Most studies were conducted between 1990 and 2015 (inclusive) and included a total of 2 995 082 glioma cases. The median patient age was 52. More than or less than9.063 years, and the mean duration of follow-up was 8.76 ± 4.433 years.

### Quantitative Synthesis

The primary aim of this study was to provide updated estimates of glioma risk among females using OCPs or receiving HRT.

#### HRT and Glioma Risk.

**Figure 2.**
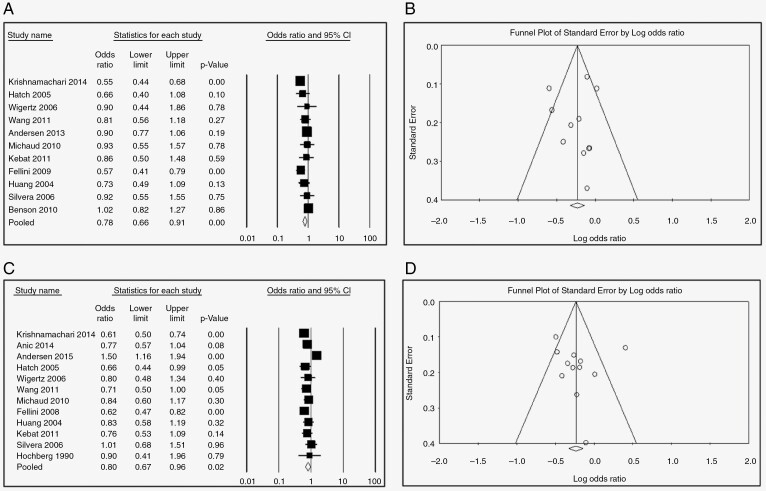
(A) Forest plots for the OR of developing Glioma after HRT regardless of the duration of use, (B) funnel plot for HRT use and glioma, (C) forest plots for the OR of developing glioma after OCP regardless of the duration of use, (D) funnel plot for OCP use.

**Figure 3.**
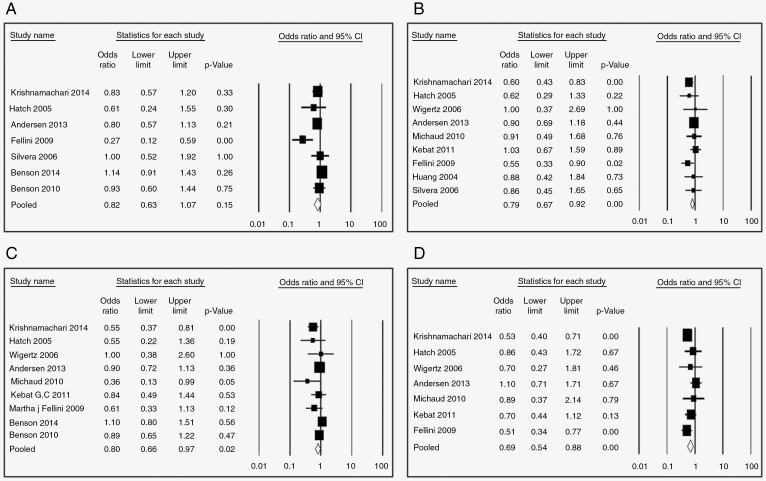
Odds ratio of developing glioma after HRT use (A) for <1 year, (B) 1–5 years, (C) 5–10 years, (D) for >10 years.

**Figure 4.**
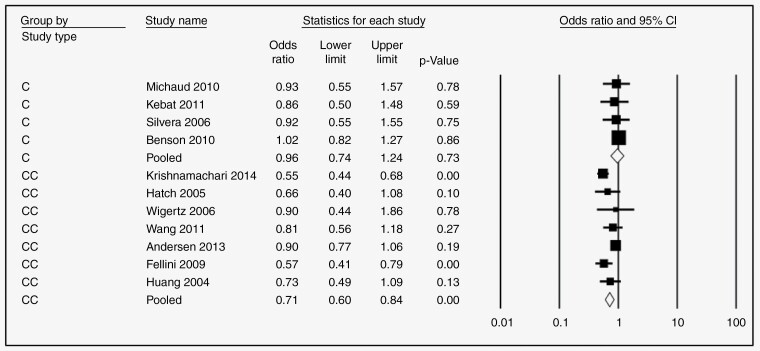
Forest plots for the OR of developing Glioma after HRT regardless of the duration of use, stratified by study type, C = cohort study, CC = case–control study.

Nine studies examined the association between HRT and glioma risk. The pooled risk estimate for users (any duration) versus never users suggests a significant protective effect (OR = 0.78, 95% CI: 0.66–0.91, *P*-value = .000, *I*^2^ = 58.08) (Figure 2A). Further, this protective effect was still significant in subgroups stratified by duration of use if > 1 year (<1 year: [OR = 0.82, 95% CI 0.63–1.07, *P* = 0.15, 58.90%] Figure 3A; 1–5 years: [OR = 0.79, 95% CI 0.67–0.92, *P* = .000, *I*^2^ = 0.13%}, Figure 3B; 5–10 years: [OR = 0.80, 95% CI 0.66–0.97, *P* = .002, *I*^2^ = 36.49%], Figure 3C; >10 years: [OR = 0.69, 95% CI 0.54–0.88, *P* = .000, *I*^2^ = 39.01%] Figure 3D). The protective effect of HRT was highly significant for case–control studies (OR = 0.71, 95% CI 0.60–0.84, *P* = .00) but not cohort studies (OR = 0.96, 95% CI 0.74–1.24, *P* = 0.73), Figure 4.

#### OCPs and Glioma Risk.

**Figure 5.**
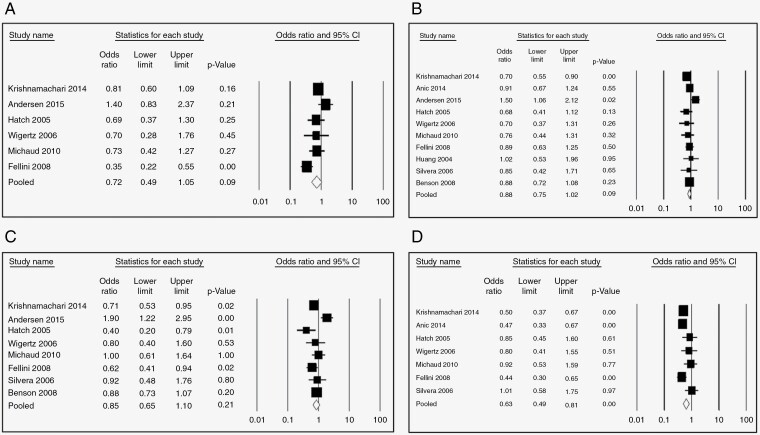
Odds ratio of developing glioma after OCP use (A) for <1 year, (B) 1–5 years, (C) 5–10 years, (D) for >10 years.

**Figure 6.**
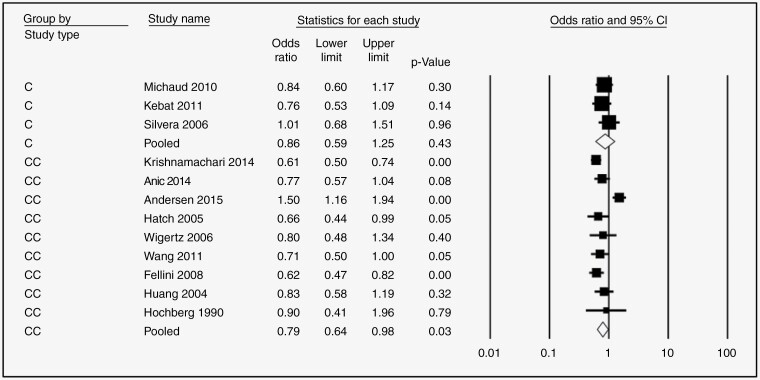
Forest plots for the OR of developing glioma after OCP regardless of the duration of use, stratified by study type, C = cohort study, CC = case–control study.

—Risk estimates for OCP ever use versus never use were reported in ten studies. The overall pooled OR was 0.80 (95% CI 0.67–0.96, *P* = .002, *I*^2^ = 70.06%, Figure 2C), again indicating significant protection. However, in subgroup analysis stratified by duration of use, only use for >10 years was significantly protective (<1 year: [OR = 0.72, 95% CI 0.49–1.050, *P* = .09, *I*^2^ = 69.95 %], Figure 5A; 1–5 years: [OR = 0.88, 95% CI 0.75–1.020, *P* = .09, *I*^2^ = 37.73], Figure 5B; 5–10 years: [OR = 0.85, 95% CI 0.65–1.10, *P* = 0.210, *I*^2^ = 67.82] Figure 5C; >10 years: [OR = 0.58, 95% CI 0.45–0.74, *P* = .000, *I*^2^ = 43.15%], Figure 5D). Like HRT, the protective effect of OCPs was significant only for case–control studies (OR = 0.79, 95% CI 0.64–0.98, *P* = .03) but not cohort studies (OR = 0.86, 95% CI 0.59–1.25, *P* = 0.43), figure 6.

### Quality Assessment and Bias

#### Risk of Bias.

Quality assessment was conducted using the NOS scale. Two studies were given a score of 5 stars, 4 studies a score of 6 stars, 5 a score of 7 stars, and the rest a score of 8 stars (all out of 9). Based on a score of 6 or higher, 15 studies (88%) were classified as high quality.

#### Sensitivity Analysis and Publication Bias.

—Omitting each study separately yielded no significant changes in OR, indicating that the results were stable and robust. Construction of a Begg’s funnel plot and Egger’s test also yielded no evidence of publication bias (Figure 2B and D). We also examined the effect of omitting the 2 studies with high risk of bias (NOS scores of 5), one a case–control study on the effects of HRT and one a case–control study examining the effects of OCPs on glioma risk,^16,17^ but again significant protection was maintained (OR = 0.76, 95% CI 0.63–0.91, *P* = .000, *I*^2^ = 55.54 and OR = 0.72, 95% CI 0.65–0.80, *P* = .000, *I*^2^ = 00.00, respectively).

## Discussion

This updated meta-analysis aimed to determine the effects of HRT and OCP on glioma risk among adult females. The pooled dataset included 12 case–control and 5 cohort studies with an overall total of 2 995 082 glioma patients. Pooled analysis revealed that prolonged OCP use (>10 years) significantly reduced the risk of developing glioma, consistent with previous findings.^10–13^ Similarly, HRT reduced the risk of developing glioma, also consistent with previous studies,^11,13^ but this protective effect required only 1 year or more of treatment. Further, sensitivity analysis in which studies with NOS score < 6 were removed (leaving only studies deemed high quality) yielded qualitatively similar results. Additional subgroup analysis revealed that the protective effects of both treatments were only significant in case–control studies. However, it is well known that case–control studies carry a higher risk of bias due to potential improper control group selection, especially for rare diseases such as glioma. For instance, using interviews or registries to identify participants with equivalent exposure can be a challenge, and in some of these case–control studies, exposure risk was taken from a proxy interviewer due to death or disability. Therefore, caution is warranted in interpreting these results, and future large-scale prospective studies are essential for confirmation.

The protective effect of HRT against glioma development is likely related to direct hormonal effects as glioma cells express steroid hormone receptors. However, Benson et al. found an increased risk of glioma among patients receiving HRT for any length of time (ever use subgroup).^9^ This contradictory finding suggests that the relationship between HRT and glioma is influenced by other factors, such as the timing, dose, type, and duration of HRT, and possibly also by individual differences in hormone metabolism. Anderson et al. also reported a significant increase in glioma risk among OCP users, particularly females taking progesterone-only therapy, and this enhanced risk was specific for glioblastoma multiforme, the most aggressive and deadly form of glioma.^16^ Several potential confounders may account for these discrepancies. Progesterone-only pills are usually prescribed for overweight women, and obesity alone has been identified as a risk factor for CNS tumors.^17^ Further, data on OCP were collected from a prescription registry initiated in 1995, and so may exclude longer-term use by older females (i.e. the sample included a disproportionate number of females <50 years old).^16^ Therefore, this result may not be applicable to older females. In fact, Hatch et al. found that OCPs reduced overall glioma risk, but stratification by age at diagnosis based on a cutoff of 50 years revealed that the protective effect was significant only in the older age group, possibly because older patients are more likely to have used more potent preparations before the 1970s.^18^

Hormone replacement therapy is prescribed more often for females with higher education and socioeconomic status. For instance, Hatch et al. found that HRT cases were better educated than controls.^18^ Similarly, Felini et al. found a greater number of low-income participants among controls in their study, although there were equal numbers of high-income earners among cases and controls.^26^ However, no stratified analysis based on income was conducted in either study. Alternatively, Benson et al. found that socioeconomic status had no effect on CNS tumor incidence, including glioma and meningioma incidence.^28^ Nonetheless, we acknowledge that an association between HRT and income or education could influence glioma incidence and thus should be included in future studies.

A previous meta-analysis by Zong et al. also found that older age at menarche was associated with a higher risk of brain tumors and glioma in particular. In addition, a longer duration of breastfeeding was associated with higher glioma risk, although with lower meningioma risk. In contrast, other reproductive factors such as menopausal status, parity, age at first birth, and age at menopause exhibited no significant association.^12^ The meta-analysis by Benson et al. also examined the influence of HRT type on CNS tumor risk and found enhanced risk among estrogen-only users, amounting to an absolute excess risk of 2/10 000 users over 5 years, while no difference in risk was found for estrogen–progesterone users.^9^ Therefore, the HRT type should also be included in future studies.

The associations of HRT and OCP exposure with lower glioma incidence both became stronger as the duration of use increased, but significant protection required only 1 year for HRT but 10 years for OCPs. These findings are in partial accord with the results of Yang et al., who found that only OCPs used for 7.5 years or more substantially reduced the risk of glioma.^10^ This difference in the effect of treatment duration between OCPs and HRT may be explained by age, as OCPs are used by premenopausal females while HRT tends to be prescribed for older females already at increased risk of glioma.

One important factor missing from some of the included studies was the particular type of glioma. This lack of specificity is concerning because glioma types may be differentially sensitive to OCP exposure. This gap may lead to false perceptions regarding risks for specific glioma types. However, gliomas are rare tumors, so stratification according to type is challenging. Other limitations of this meta-analysis include the absence of age stratification in some studies. While the majority of studies found reduced glioma risk among exogenous hormone users, especially after prolonged use, the pooled result is inconsistent with some individual studies. Thus, larger-scale prospective studies considering possible confounders such as age at menarche, age at menopause, parity, breastfeeding history, age during treatment, hormone type(s), and dose among others are required to establish more accurate associations with glioma risk.

A funnel plot revealed no signs of publication bias. However, publication bias is a potential limitation of all meta-analyses as it is well known that negative results are often not published. Finally, the source of funding can be a potential source of bias, and 2 studies did not mention the source of funding.

## Conclusion

This meta-analysis suggests an association between HRT for at least 1 year and OCP for at least 10 years and a reduction in the overall risk of glioma among adult females. However, additional research is needed to elucidate the mechanisms underlying this protective effect. Such information could help in the development of therapeutic applications for the prevention or treatment of glioma.

## Data Availability

The datasets generated and analyzed during the current study are available from the corresponding author on reasonable request.
